# Ectopic thyroid tissue in the breast: A case report

**DOI:** 10.1515/biol-2022-0628

**Published:** 2023-06-14

**Authors:** Zheng Gong, Yan Yang, Zegang Liu

**Affiliations:** Department of Thoracic Surgery, The Affiliated Hospital of Yunnan University, Kunming, China; Department of General Surgery, 920th Hospital of Joint Logistics Support Force, PLA, Kunming, China

**Keywords:** ectopic thyroid tissue, breast, embryology, case report

## Abstract

Ectopic thyroid tissue is a rare condition manifested as the appearance of thyroid tissue outside the thyroid gland. Here, we report a case of ectopic thyroid tissue in the breast. A 48-year-old Chinese woman who was diagnosed with breast cancer received modified radical mastectomy. A thyroid tissue was found on subsequent pathological examination. The ectopic thyroid tissue was confirmed by immunohistochemistry staining of thyroid biomarkers, including thyroglobulin, thyroid transcription factor-1, and thyroid peroxidase. Currently, abnormal thyroid anlage descent is the main theory to explain ectopic thyroid tissue, especially lingual thyroid. However, it is far-fetched to explain the pathogenesis of ectopic thyroid tissues existed in organs or tissues far from thyroid such as iris, cardiac, pulmonary, duodenal, adrenal, and vertebral. Here, we reviewed the previous cases of ectopic thyroid tissue in breast and proposed a “entoderm migration” theory to explain distant ectopic thyroid tissues based on embryonic development perspective.

## 1 Introduction

Ectopic thyroid tissue is a rare condition accounts about 1 case per 100,000 to 300,000 persons, and its pathogenesis is not clear [1]. The majority of patients with thyroid ectopia are asymptomatic and have been considered benign. Since thyroid sarcomas tend to spread to the breast and thoracic cavity, it is important to confirm whether there are thyroid cancer especially thyroid sarcomas when an ectopic thyroid tissue is diagnosed. The current theory considered that abnormal migration of thyroid anlage during the embryonic period resulting ectopic thyroid tissue especially lingual thyroid [2]. However, this theory is defective to explain thyroid tissues existed in the areas which is far from the thyroid anlage migration path. In this case report, we presented a rare case of breast ectopic thyroid tissue and proposed a “entoderm migration” theory to interpret its pathogenesis. We also discussed differential diagnosis between thyroid ectopia and thyroid sarcomas and reviewed the literature associated with ectopic thyroid tissue.

## 2 Case presentation

**Figure 1:**
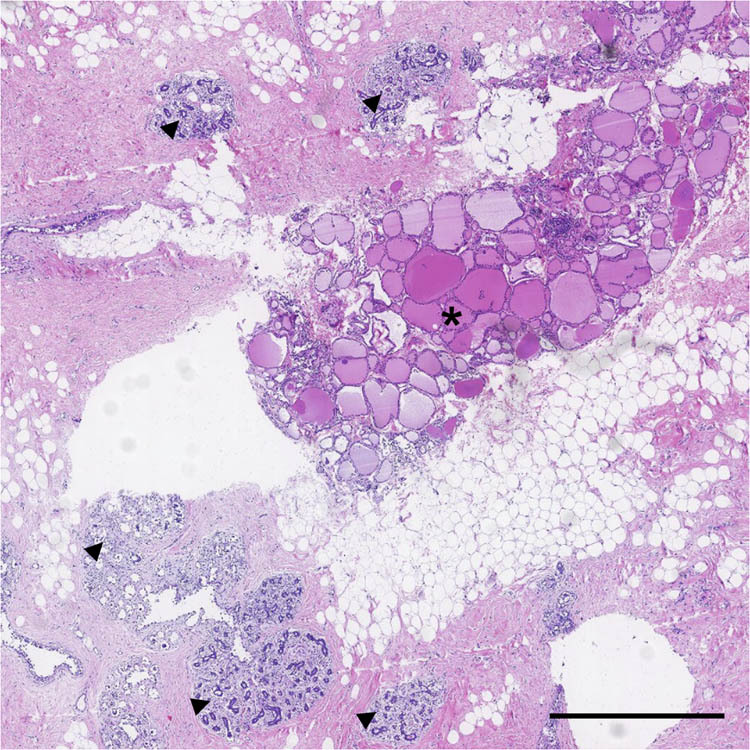
Ectopic thyroid tissue found in breast. Hematoxylin and eosin (HE) staining shows thyroid-like structure (*) and breast structure (triangles). The scale bar is 1 mm.

**Figure 2:**
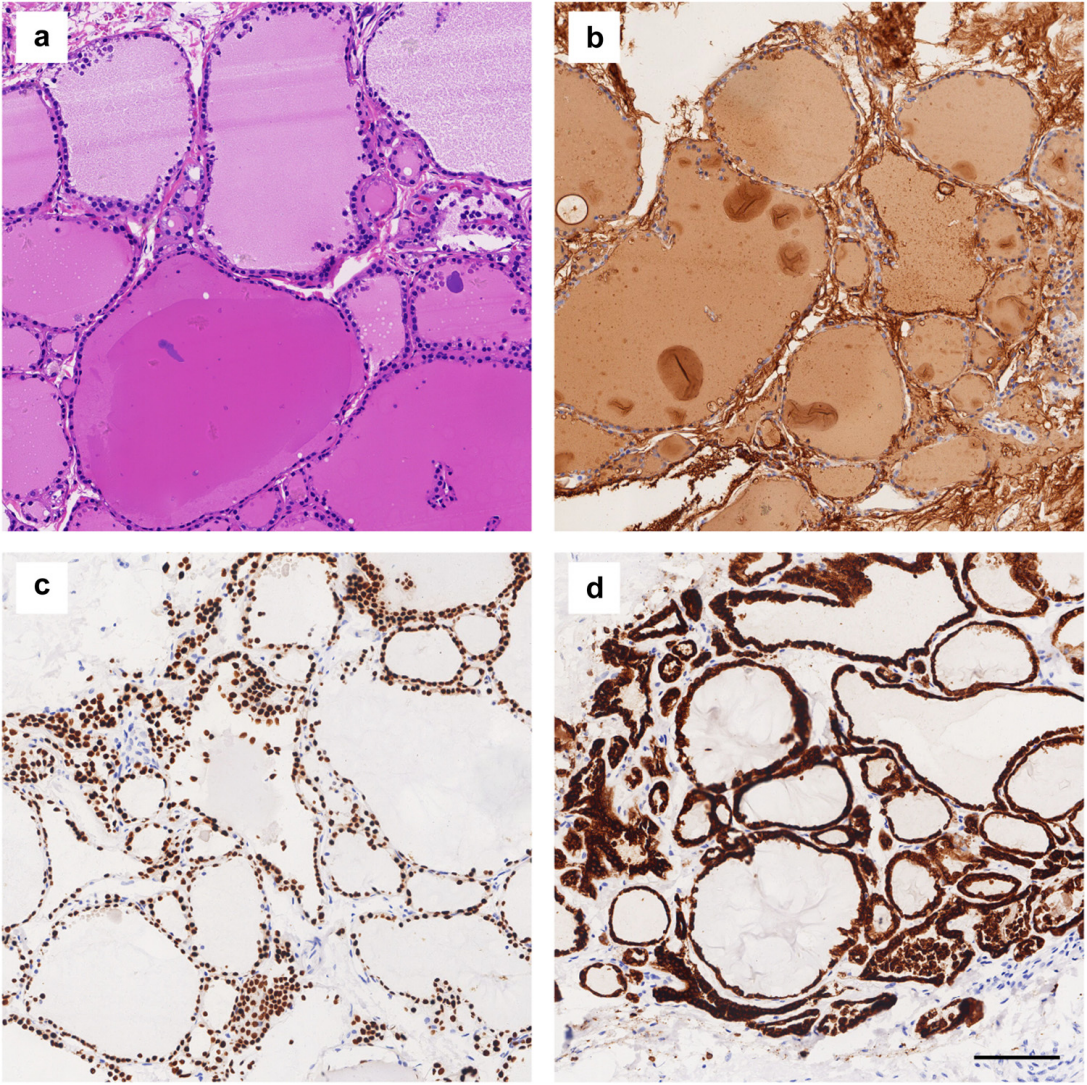
IHC staining of ectopic thyroid tissue. (a) HE staining shows TFCs and colloids. (b) Positive immunohistochemistry staining of TG. (c) Positive staining of TTF-1. (d) Positive staining of TPO. The scale bar is 100 μm.


In July 2018, a 48-year-old Chinese woman came to our hospital searching for further breast cancer therapy. Before admitted to our hospital, the patient had accepted treatment in other hospital, because of left breast mass. In the previous hospital, she underwent a surgical resection of left breast mass and post-operation pathological examination confirmed left breast invasive ductal carcinoma. For further treatment, she came to our hospital. The patient had no history of other malignancy, smoking, or drinking history. She had no family history of any cancer or thyroid diseases. When she was admitted to our hospital, there were no inflammatory signs and no anemia or dyspnea. Physical examination indicated a surgical incision in the outer lower quadrant of left breast, 5.6 cm away from nipple. There were also touchable enlarged lymph nodes in the left axillary. The thyroid ultrasound scan did not find any nodules. The thyroid function tests were normal: thyrotropin (TSH) 1.51 µIU/mL (0.49–4.91 µIU/mL), free thyroxine (FT4) 10.49 pmol/L (7.64–16.03 pmol/L), and free triiodothyronine (FT3) 4.76 pmol/L (3.28–6.47 pmol/L). Because the patient had been diagnosed as breast carcinoma by pathological examination, she received modified radical mastectomy in our hospital. After surgery, the excised breast tissue went on pathological examination to confirm the pathological type of tumor. A touchable hard area in the outer upper quadrant was found. The transection of this hard area showed colors of sallow, different from the neighboring tissue. To further explore the histological characteristics, this area was sliced and performed HE staining. The HE staining image (Figure 1) showed mammary gland structures including glandular acinus and cystic hyperplasia, which accompanied usual ductal hyperplasia and columnar cell lesions. Moreover, the thyroid-like structures were found besides the breast tissue and was precisely the tissue of the color of sallow which was mentioned above. The thyroid tissue regions had obvious thyroid-like follicles that containing pink colloid and were surrounded by cells with similar character of thyroid follicular epithelial cells (Figure 2a). There were no cancer cells found in all slices. To explore whether the regions were thyroid tissues, immunohistochemistry (IHC) staining was performed. IHC staining indicated that thyroglobulin (TG) distributing in thyroid follicles, suggesting the origin of thyroid tissues (Figure 2b). Thyroid transcription factor-1 (TTF-1) expressed in cells which surrounding follicles revealed follicular cells (Figure 2c). Thyroid peroxidase (TPO) existed in the apical membrane of the thyrocyte further confirmed ectopic thyroid gland (Figure 2d). Negative staining of biomarkers such as Ki67 and Galectin-3 excluded possibility of thyroid cancer (data not shown).

**Informed consent:** Informed consent has been obtained from all individuals included in this study.
**Ethical approval:** The research related to human use has been complied with all the relevant national regulations, institutional policies and in accordance with the tenets of the Helsinki Declaration, and has been approved by the authors’ institutional review board or equivalent committee.

## 3 Discussion

An ectopic thyroid is first described by Hickman in 1869 and is defined as thyroid tissue that is not located in the second to fourth tracheal cartilages [3]. Ectopic thyroid is a rare condition and has been reported in tracheal, submandibular, lateral cervical region, etc. [4,5,6]. However, there are only a few reports about ectopic thyroid tissue in breast. Teixeria et al. first reported a case of ectopic thyroid tissue in breast [7]. In this case, the 85-year-old woman with surgical history of total thyroidectomy was diagnosed with ectopic thyroid in breast and received surgical excision. The author raised the question of whether the mild hyperthyroid status was an iatrogenic hyperthyroidism or a result of functioning residual ectopic thyroid tissue. Based on our case here, although ectopic thyroid tissue existed in breast, the thyroid function of the patient was normal. Ectopic thyroid tissue in the breast seems not affect thyroid function. Joseph et al. reported an ectopic intrathoracic thyroid tissue with breast carcinoma [8]. They proposed that it is important to confirm the nature of intrathoracic mass, because the treatment plan should be entirely changed if the mass is a metastatic lymph node. It has been reported that thyroid sarcomas tend to spread to the breast, so it is crucial to distinguish ectopic thyroid tissue from thyroid sarcoma. The diagnosis of thyroid sarcoma relies mainly on IHC staining. Thyroid follicular dendritic cell sarcoma (FDCS) is a malignancy originates from follicular dendritic cells. Seyed-Alagheband et al. reported that this tumor showed positive dendritic cell markers including CD21, CD23, CD35, CD45, and clusterin. Moreover, CD21 and CD35 have higher sensitivity and specificity [9]. Besides FDCS, primary thyroid synovial cell sarcoma (SVS) is another tumor need to be considered. Primary thyroid SVS is a malignancy with poor biological behavior. The tumor is aggressive and shows evidence of distant metastasis. The expression of EMA, CK17, CK19, BCL-2, and TLE1 can help to confirm Primary thyroid SVS [10].

The etiology of ectopic thyroid tissue is not fully understood. Moon et al. reported an ectopic thyroid tissue in breast and indicated that it is difficult to explain this condition by common abnormal migration theory, because breast is distant from the path of thyroid gland embryological development [11]. The thyroid gland contains two cell types, which are thyroid follicular cells (TFCs) and C cells. During embryo development, these cells come from different places of blastoderm and develop in different period [12]. As is known, thyroid gland development mainly contains three processes [13]. First, the thyroid anlage appears at a 3-week human embryo. Second, the anlage migrates to the thyroid place during 6–7 weeks. Third, the ultimobranchial body migrates to thyroid gland and differentiates to C cells and then the development of thyroid gland is finished. To understand the pathogenesis, most research and theories focused on the migration path of thyroid anlage and could explain ectopic thyroid gland around thyroglossal duct. But the migration theory hardly gives explanation to distant ectopic thyroid, such as esophageal thyroid [14].

The embryonic development is a complex and precisely regulated process. It involves many biological activities including cell migration and differentiation. For example, primitive streak cells move away from the original positions and develop into endoderm and mesoderm. In the period of 3-week embryo, paraxial mesoderm develops into somites and begins to differentiate in the period of 4-week embryo accompanied by cell position changing. Through the analysis of embryonic development, we found that the time of thyroid anlage formation and migration overlaps with somite formation and differentiation. Hence, we proposed a theory to explain distant ectopic thyroid. The thyroid anlage appears in entoderm and forms a small hole toward the mesoderm. At this stage, two cell layers of entoderm and mesoderm are very closed in space, giving the possibility for abnormal cell exchanging. During cardiovascular system development, dorsal aorta and primitive heart tube are close to endoderm in space and may explain why the thyroid tissue could occur in aorta or heart. In the period of 3-week embryo, some cells from thyroid anlage may abnormally leave from the position and migrate into mesoderm and mix with somite cells. Then, the thyroid anlage cells will move to different areas following somite developmental processes. Also, a small number of thyroid anlage cells could move through mesoderm to ectoderm, which will develop into epidermis and breast gland. Considering the probability of reaching the ectoderm through the mesoderm is lower than that of directly reaching the mesoderm, the incidence of breast ectopic thyroid tissue is lower than other parts of the body.

This theory could explain the etiology of breast ectopic thyroid tissue and other types of distant ectopic thyroid. Because thyroid anlage cells in entoderm migrate to different positions and move along with another structure, ectopic thyroid will exist in tracheal, lateral cervical, axillary, carotid, duodenal, vertebral body, and other distant places [15,16,17].

In addition to the correlation between breast and ectopic thyroid in embryonic development, some clinical researches have indicated relationships in breast cancer and thyroid cancer [18,19]. It has been reported that breast cancer patient is more likely occurred by thyroid cancer [20]. Thus, more researches need to be done to clarify the molecular mechanisms of ectopic thyroid tissue in the breast.

## 4 Conclusion

Overall, we reported a rare case of ectopic thyroid tissue in breast, reviewed the literature, and indicated the importance of distinguishing from sarcomas. More importantly, we proposed the “entoderm migration” theory to explain the etiology of distant thyroid ectopia. This article raises awareness of the causes and diagnosis of ectopic thyroid tissues.
